# Protein A-Mouse Acidic Mammalian Chitinase-V5-His Expressed in Periplasmic Space of *Escherichia coli* Possesses Chitinase Functions Comparable to CHO-Expressed Protein

**DOI:** 10.1371/journal.pone.0078669

**Published:** 2013-11-11

**Authors:** Akinori Kashimura, Kazuaki Okawa, Kotarou Ishikawa, Yuta Kida, Kokoro Iwabuchi, Yudai Matsushima, Masayoshi Sakaguchi, Yasusato Sugahara, Fumitaka Oyama

**Affiliations:** 1 Department of Applied Chemistry, Kogakuin University, Hachioji, Tokyo, Japan; University of Delhi, India

## Abstract

Acidic mammalian chitinase (AMCase) has been shown to be associated with asthma in mouse models, allergic inflammation and food processing. Here, we describe an *E. coli*-expression system that allows for the periplasmic production of active AMCase fused to Protein A at the N-terminus and V5 epitope and (His)_6_ tag (V5-His) at the C-terminus (Protein A-AMCase-V5-His) in *E. coli*. The mouse AMCase cDNA was cloned into the vector pEZZ18, which is an expression vector containing the *Staphylococcus* Protein A promoter, with the signal sequence and truncated form of Protein A for extracellular expression in *E. coli*. Most of the Protein A-AMCase-V5-His was present in the periplasmic space with chitinolytic activity, which was measured using a chromogenic substrate, 4-nitrophenyl *N,N*′-diacetyl-β-D-chitobioside. The Protein A-AMCase-V5-His was purified from periplasmic fractions using an IgG Sepharose column followed by a Ni Sepharose chromatography. The recombinant protein showed a robust peak of activity with a maximum observed activity at pH 2.0, where an optimal temperature was 54°C. When this protein was preincubated between pH 1.0 and pH 11.0 on ice for 1 h, full chitinolytic activity was retained. This protein was also heat-stable till 54°C, both at pH 2.0 and 7.0. The chitinolytic activity of the recombinant AMCase against 4-nitrophenyl *N,N*′-diacetyl-β-D-chitobioside was comparable to the CHO-expressed AMCase. Furthermore, the recombinant AMCase bound to chitin beads, cleaved colloidal chitin and released mainly *N,N′*-diacetylchitobiose fragments. Thus, the *E. coli*-expressed Protein A-mouse AMCase-V5-His fusion protein possesses chitinase functions comparable to the CHO-expressed AMCase. This recombinant protein can be used to elucidate detailed biomedical functions of the mouse AMCase.

## Introduction

Chitin is a polymer of (β-1.4)-linked *N*-acetyl-D-glucosamine, which is an integral component of the exoskeleton of crustaceans and insects, the microfilarial sheaths of parasites and the cell walls in fungus [1], [2].

Chitinase (EC 3.2.1.14) hydrolyzes chitin. It is an important enzyme that is responsible for chitin metabolism in a wide range of organisms, including bacteria, fungi, nematodes and arthropod [2], [3]. Although mammals do not produce their own chitin, two active chitinases, chitotriosidase (Chit1) and acidic mammalian chitinase (AMCase), have been identified in mice and humans [3]. Both enzymes show sequence homology to bacterial chitinases and belong to the family 18 of glycoside hydrolases [4], [5] [the carbohydrate active enzymes (CAZy) database, http://www.cazy.org/], which also includes chitinase-like proteins that are structurally related to chitinases but lack chitinolytic activity [3], [6], [7].

Marked elevation of Chit1 activity has been reported in Gaucher disease, which is an autosomal recessive lysosomal storage disorder [8], [9]. Chit1 was the first mammalian chitinase to be purified and cloned [10], [11]. The physiological role of Chit1 is the defense against chitin-containing pathogens [12]. However, a recessively inherited deficiency in Chit1 activity is commonly observed in Caucasians, suggesting that Chit1 acts as a sole chitinase, performing a defensive function under normal circumstances [13].

Acidic mammalian chitinase (AMCase), another mammalian chitinolytic enzyme, was discovered to perform this type of compensatory role and was named for its acidic isoelectric point [14]. AMCase is a 50 kDa enzyme that is expressed primarily in the mouse stomach and lung [14], [15]. Unlike other chitinases, which are inactive at low pH, AMCase can withstand a low pH environment. Mouse AMCase has been shown to be most active at pH 2.0 and is acid-stable [14].

AMCase has attracted considerable attention due to its increased expression under pathological conditions. Significant increases of AMCase mRNA and protein were detected in an induced asthma mouse model [16]. Polymorphisms and haplotypes of AMCase are associated with bronchial asthma in humans [17], [18]. Furthermore, AMCase expression is increased by antigen-induced mouse models of allergic lung inflammation [19]. Recently, we found that AMCase mRNA is synthesized in the mouse stomach at extraordinarily high levels, which are comparable to pepsinogen C, a major digestive enzyme in the gastric juice [20], [21]. Thus, AMCase may play an important role in asthma, immune response and food processing. Little is known, however, about the pathophysiological functions of AMCase in mice and humans.

The biochemical characterization of chitinases requires large quantities of purified protein. Currently, efforts to characterize these proteins structurally and biochemically rely on mammalian and insect cell expression systems and expression in *E. coli* [14], [16], [18], [19], [22]–[27]. Here, we describe an *E. coli* expression system that allows for the production of an active AMCase fused to Protein A, V5 epitope and (His)_6_ tag (Protein A-AMCase-V5-His). We used pEZZ18 [28], which is an extracellular expression vector containing the *Staphylococcus aureus* Protein A promoter, with the signal sequence and truncated form of Protein A (the synthetic ZZ domain). Because AMCase is a secretory protein and is acid-stable [14], the expressed fusion protein was expected to be secreted into culture medium. However, we found that a large portion of the fusion protein was present in the periplasmic fraction of *E. coli*. The *E. coli*-produced Protein A-mouse AMCase-V5-His fusion protein had both chitinolytic and chitin-binding activities that were comparable to mammalian cultured cell-expressed AMCase.

## Materials and Methods

### Mammalian Expression Vector

**Figure 1:**
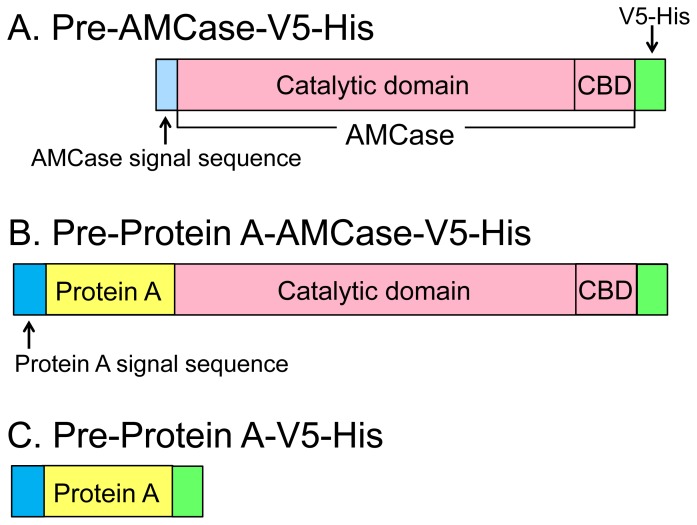
The schematic representations of the CHO- and *E. coli*-expressed mouse AMCase fusion proteins. Mouse AMCase is a secreted protein with a molecular mass of approximately 50-terminal catalytic domain and a C-terminal chitin-binding domain (CBD). (A) CHO-expressed pre-AMCase-V5-His. (B) *E. coli*-expressed pre-Protein A-AMCase-V5-His. (C) *E. coli*-expressed pre-Protein A-V5-His. The *E. coli*-produced proteins contain the affinity tail of Protein A at the N-terminus. CHO- and *E. coli*-recombinant proteins contain V5-His at the C-terminus. Newly synthesized recombinant proteins contain the AMCase signal sequence (A) or Protein A signal sequence (B and C).

We used mouse stomach total RNA from the Mouse Total RNA Master Panel (Clontech Laboratories) and reverse transcribed the RNA into cDNA, as previously described [20]. To express mouse AMCase precursor-V5-His (pre-AMCase-V5-His, Figure 1A), AMCase cDNA (GenBank accession number AK160173.1, nucleotides 6∼1435) was amplified from the mouse stomach cDNA by PCR using KOD Plus DNA polymerase (Toyobo) and oligonucleotide primers (Sigma-Aldrich Life Science Japan) anchored with the restriction sites for EcoRI and XhoI (**Table S1**). The forward primer (EcoRI-pre-AMCase-Fw) contains 6 bases long EcoRI recognition sequence (underlined) and 25 bases long AMCase sequence corresponded to nucleotides 6∼30 of the AMCase cDNA (**Table S1**). The reverse primer (XhoI-pre-AMCase-Rv) contains the XhoI recognition sequence (underlined) and is complementary to nucleotides 1413∼1435 of the AMCase cDNA and both primers contain the 4∼5 bases long extra nucleotides (boldfaced) to cleavage close to the end of the amplified the cDNAs by restriction enzyme efficiently (**Table S1**). The amplified DNA contains one EcoRI and one XhoI sites anchored with the PCR primers. The PCR product was purified using the Wizard SV Gel and PCR Clean-Up System (Promega) and then digested with EcoRI and XhoI. The DNA fragment was purified using 1.5% agarose gel electrophoresis and then purified using the Clean-Up System and subcloned into a similarly digested pcDNA3.1/V5-His C vector (Invitrogen). We designed the reverse primer, which is in frame with the carboxyl terminal region of V5-His of pcDNA3.1/V5-His C vector. The entire nucleotide sequence of the resulting plasmid DNA (the pcDNA3.1/pre-AMCase-V5-His) was confirmed by sequencing using the ABI PRISM Big-Dye Terminator v3.1 Cycle Sequencing Kit and the 3130 Genetic Analyzer instrument (Applied Biosystems).

### *E. coli* Expression Vector

The mature AMCase-V5-His cDNA region without its signal sequence was amplified from the pcDNA3.1/pre-AMCase-V5-His by PCR using KOD Plus DNA polymerase and primers anchored with EcoRI and SalI restriction sites. The forward primer (EcoRI-mature-AMCase-Fw) contains EcoRI recognition sequence, which is in frame with the carboxyl terminal region of Protein A, and nucleotides 80∼103 of the AMCase cDNA (**Table S1**). The reverse primer (SalI-pcDNA BGH-Rv) contains SalI recognition sequence and is complementary to nucleotides 1080∼1106 of pcDNA3.1/V5-His C vector (http://tools.invitrogen.com/content/sfs/vectors/pcdna3_1v5hisc_seq.txt). Both primers contain the 4∼5 bases long extra nucleotides at their 5′ ends as described above (**Table S1**). The amplified DNA was digested with EcoRI and SalI and subcloned into a similarly digested pEZZ18 (GE Healthcare) [28]. The plasmid containing the cDNA insert was selected and sequenced as described above. By using the plasmid DNA (the pEZZ18/pre-Protein A-AMCase-V5-His), we transformed *E. coli* BL21 (DE3) (Novagen) to express pre-Protein A-AMCase-V5-His (Figure 1B). As a control, we also constructed pre-Protein A-V5-His (Figure 1C).

### The Preparation of the Recombinant Protein from the Medium, Periplasmic Space and Soluble Fractions of *E.* *coli*

Transformed *E. coli* BL21 (DE3) strains were grown in 1 L LB medium containing 100 µg/mL ampicillin at 37°C for 18 h. Cells were harvested by centrifugation at 5,000 × *g* for 20 min at 4°C, and the supernatants were pooled (culture medium fraction). Cells were suspended in 30 mL of 20 mM Tris-HCl (pH 7.6) containing 20% sucrose (w/v), 0.5 mM EDTA and a protease inhibitor (Complete, Roche), incubated on ice for 30 min, and then centrifuged at 15,000 × *g* for 15 min at 4°C. After the addition of 120 mL of 20 mM Tris-HCl (pH 7.6) containing the protease inhibitor, the cells were further incubated on ice for 30 min and centrifuged at 15,000 × *g* for 15 min at 4°C. The supernatants were combined (periplasmic space 1/osmotic shock fraction). The resulting cell pellet was suspended again in 30 mL of 20 mM Tris-HCl (pH 7.6) containing 20% sucrose (w/v), 0.5 mM EDTA, lysozyme (3 mg/mL, Wako Pure Chemicals) and a protease inhibitor. The sample was then incubated at 37°C for 15 min and centrifuged at 15,000 × *g* for 15 min at 4°C. After the addition of 120 mL of 20 mM Tris-HCl (pH 7.6) containing a protease inhibitor, the cells were further incubated on ice for 30 min and centrifuged at 15,000 × *g* for 15 min at 4°C. The supernatants were pooled. To reduce the viscosity, the solution was briefly sonicated and NaCl was added at a final concentration of 150 mM (periplasmic space 2/lysozyme fraction). The resulting cell pellet was suspended in 20 mM Tris-HCl (pH 7.6), 150 mM NaCl and sonicated and centrifuged at 15,000 × *g* for 15 min at 4°C and the supernatant was pooled (cytoplasmic soluble fraction).

These fractions were applied to an IgG Sepharose (GE Healthcare) column equilibrated with TS buffer [20 mM Tris-HCl (pH 7.6), 150 mM NaCl and protease inhibitor]. After extensive washing, the bound fusion protein was eluted with 0.1 M Gly-HCl (pH 2.5) followed by neutralization with 1 M Tris-HCl (pH 7.6), and the active fractions were desalted with PD10 (GE Healthcare) equilibrated with the TS buffer.

### The Separation of the Recombinant Protein from the Insoluble Fractions by Ni Sepharose

It is known that some parts of the His-tagged proteins can be in insoluble form and collect as a pellet after the centrifugation of the disrupted cells [29]. The insoluble fraction was solubilized in 8 M urea in 20 mM Tris-HCl (pH 7.6) solution containing protease inhibitor for 30 min at 4°C. The samples were then centrifuged at 15,000 × *g* for 15 min at 4°C and the supernatants were pooled (solubilized “insoluble fraction”). Refolding and purification of the recombinant protein were then performed on a Ni Sepharose column (GE Healthcare). The solubilized fraction was applied to a Ni Sepharose column and the His-tagged protein was captured. The column was washed using 10-column volumes of 8 M urea in 20 mM Tris-HCl (pH 7.6). Then, the resin was washed with 10-column volumes of 0.05 M imidazole, 0.5 M NaCl in 20 mM Tris-HCl (pH 7.6). Bound proteins were eluted with 0.5 M imidazole, 0.5 M NaCl in 20 mM Tris-HCl (pH 7.6) and desalted, as described above.

### The Purification of Protein A-AMCase-V5-His by IgG Sepharose Followed by Ni Sepharose Chromatographies

The Protein A-AMCase-V5-His from the periplasmic fractions was purified by IgG Sepharose using the N-terminal Protein A and Ni Sepharose using C-terminal His-tag. The periplasmic fraction was prepared as described above and MgSO_4_ was added to a final concentration of 2 mM. The periplasmic fraction was applied to IgG Sepharose resin. The bound protein was eluted with 0.1 M Gly-HCl (pH 2.5) followed by neutralization with 1 M Tris-HCl (pH 7.6). The peak fractions were pooled and applied to Ni Sepharose resin. The column was washed with 10-column volumes of 50 mM imidazole, 0.5 M NaCl in 20 mM Tris-HCl (pH 7.6) and proteins were eluted with 0.5 M imidazole, 0.5 M NaCl in 20 mM Tris-HCl (pH 7.6) and desalted as described above. To avoid additional freeze/thaw cycles, we divided the purified enzyme into aliquots and these were stored in a freezer at −80°C.

### The Transient Expression in CHO Cells and the Purification of AMCase-V5-His from CHO Culture Medium

CHO-K1 cells (CCL61, ATCC) were regularly maintained in Minimum Essential Medium (Invitrogen) supplemented with 10% fetal bovine serum (Biowest). CHO cells were transfected with the pcDNA3.1/AMCase-V5-His expression plasmid using Lipofectamine Plus transfection reagent (Invitrogen), according to the manufacturer’s instruction. After 48 h, the cell culture media was removed from the cells. Secreted AMCase was bound to Ni Sepharose resin equilibrated with 0.5 M NaCl in 20 mM Tris-HCl (pH 7.6). Bound AMCase was eluted with 0.5 M imidazole, 0.5 M NaCl in 20 mM Tris-HCl (pH 7.6) and desalted with PD MidiTrap G-25 (GE Healthcare) equilibrated with the TS buffer.

### Determination of Protein Concentration, SDS-polyacrylamide Gel Electrophoresis and Western Blotting

Protein concentrations were determined by the Bradford Protein Assay (Bio-Rad) [30] using the BioPhotometer Plus UV/Vis photometer (Eppendorf), with bovine serum albumin as a standard. The protein fractions that were obtained as described above were analyzed using standard SDS-polyacrylamide gel electrophoresis (PAGE) [31]. The proteins in the gel were visualized by staining with Coomassie Blue R-250 (Sigma-Aldrich). Separated proteins were electrophoretically transferred to a PVDF (polyvinylidene fluoride) membrane (Immobilon-P, Millipore), which was incubated with an anti-V5-HRP monoclonal antibody (Invitrogen). Bound antibodies were detected using Immobilon Western Chemiluminescent HRP Substrate (Millipore). The immunoblots were analyzed using the Luminescent Image Analyzer (ImageQuant LAS 4000, GE Healthcare).

### Chitinase Enzymatic Assays

Chitinolytic activity was determined using the synthetic chromogenic substrate, 4-nitrophenyl *N,N*′-diacetyl-β-D-chitobioside (Sigma-Aldrich), at a concentration of 200 µM. Each reaction was done in triplicate. All enzymatic reactions for the determination of optimum pH and temperature were conducted in a volume of 50 µL containing *E. coli*- or CHO-expressed protein in McIlvaine’s buffer [14] or 0.1 M Gly-HCl buffer. Reactions for optimum pH and kinetic assays were conducted for 30 min at 37°C. Reactions were halted with the addition of 20 µL of 1 M sodium carbonate to the reaction mixture. The absorbance of the liberated 4-nitrophenolate ion was measured at 405 nm. A molar extinction coefficient for 4-nitrophenol of 17,700 M^−1^ cm^−1^ was used in the calculations. One enzyme unit (U) was defined as 1 µmol of 4-nitrophenol released from 4-nitrophenyl *N,N*′-diacetyl-β-D-chitobioside per min at 37°C in Gly-HCl buffer (pH 2.0).

### The Effects of pH and Temperature on Chitinase Activity

For the determination of the optimal pH, the chitinase activity was investigated by incubating the enzyme with 4-nitrophenyl *N,N*′-diacetyl-β-D-chitobioside as a substrate in McIlvaine’s buffer (0.1 M citric acid and 0.2 M Na_2_HPO_4_; pH 2.0 to pH 8.0) or 0.1 M Gly-HCl buffer (pH 1.0 to pH 4.0) at 37°C for 30 min. For measuring the optimal temperature, chitinase activity was assayed between 30°C and 60°C in 0.1 M Gly-HCl buffer (pH 2.0).

For the determination of the pH stability, samples were incubated for 1 h on ice in 0.1 M Gly-HCl buffer (pH 1.0 to pH 4.0), McIlvaine’s buffer (pH 2.0 to pH 8.0), Clark and Lubs buffer (0.1 M KCl, 0.1 M H_3_BO_3_ and 0.1 M NaOH; pH 8.0 to pH 10.0) and carbonate buffer (0.05 M NaHCO_3_ and 0.1 M NaOH; pH 10.0 to pH 11.0). After the pre-incubation at the indicated pH, the residual activity was analyzed at pH 2.0 in 0.1 M Gly-HCl buffer, as described above.

For heat stability measurement, samples were incubated at pH 2.0 or at pH 7.0 in McIlvaine’s buffer for 20 min between 30°C and 58°C. After cooling on ice, the residual activity was measured at pH 2.0 in 0.1 M Gly-HCl buffer, as described above.

### Chitin Binding Assay

After washing the chitin beads (50 µL, New England Biolabs) with the binding buffer [0.5 M NaCl in 20 mM Tris-HCl (pH 7.6)] three times, CHO-expressed AMCase-V5-His, *E. coli-*produced Protein A-AMCase-V5-His or Protein A-V5-His (control protein) were added to the resin, and the suspension was rotated gently at 4°C for 1 h to bind the proteins to the chitin beads. The suspension was centrifuged at 2,000 × *g* for 5 min. The supernatant was pooled as unbound fractions and analyzed by Western blot using an anti-V5 epitope antibody. The chitin slurry was suspended in the binding buffer and was washed five times. Laemmli SDS sample buffer [31] was added to the slurry, and the mixture was heated at 95°C for 5 min and analyzed by Western blot.

### The Degradation of Colloidal Chitin by *E. coli-* or CHO-expressed Mouse AMCase

Colloidal chitin was prepared from shrimp shell chitin (Sigma-Aldrich), as described previously, and used as a substrate to determine the chitinase activity [14]. All enzymatic reactions using colloidal chitin (at a final concentration of 1 mg/mL) as a substrate were carried out in a volume of 50 µL containing *E. coli*- or CHO-expressed protein in 0.1 M Gly-HCl buffer (pH 2.0). Reactions were kept for 1 h at 37°C. The chitin fragments generated by recombinant AMCase proteins were labeled covalently at their reducing end groups with the fluorophore 8-aminonaphthalene-1,3,6-trisulphonic acid (ANTS, Sigma-Aldrich), and the resulting fluorescent derivatives were separated by high-resolution PAGE, as described by Jackson [32]. *N*-acetyl chitooligoaccharides (Seikagaku Corporation) were used as a standard.

## Results

### The Construction of Recombinant Mouse AMCase Expression Plasmids

The schematic representations of the recombinant mouse AMCase proteins are shown in Figure 1. Mouse AMCase is a secretory protein with a molecular mass of approximately 50 kDa, which contains an N-terminal catalytic domain and a C-terminal chitin-binding domain (CBD) [14]. For expression in CHO cells, the entire coding region of AMCase precursor (pre-AMCase) cDNA was subcloned into the mammalian expression vector pcDNA3.1/V5-His C to produce pre-AMCase-V5-His with a signal sequence at the N-terminal region (Figure 1A and **Figure S1A**). Expression of this cDNA in CHO cells led to the secretion of the mature AMCase-V5-His into culture medium (**Figure S1B**).

For the production in *E. coli*, the mature AMCase-V5-His cDNA region was ligated with pEZZ18 [28], which contained the signal sequence of *Staphylococcus* Protein A to express pre-Protein A-AMCase-V5-His (Figure 1B and **Figure S2A**). Expression of this cDNA in *E. coli* cells led to the secretion of the mature Protein A-AMCase-V5-His directly into the cell culture medium (**Figure S2B**). We also constructed pre-Protein A-V5-His lacking the AMCase region as a control protein (Figure 1C and **Figure S2C**). Similarly, pre-Protein A-V5-His should be processed into mature Protein A-V5-His (**Figure S2D**). Thus, the *E. coli*-expressed recombinant protein contains the affinity tail of Protein A at the N-terminus when compared with CHO-expressed protein (Figure 1, **Figure S1** and **Figure S2**).

The fusion proteins can be purified either by Ni Sepharose using the His-tag (Figure 1A∼C) or IgG Sepharose using Protein A (Figure 1B and C). We detected the recombinant proteins by measuring chitinolytic activity, using 4-nitrophenyl *N,N*′-diacetyl-β-D-chitobioside as a substrate, and by Western blotting, using an anti-V5 epitope antibody.

### The Distribution of the Recombinant Fusion Protein in the Culture Medium and Intracellular Fractions in *E. coli*

The pEZZ18/pre-Protein A-AMCase-V5-His plasmid (Figure 1B and **Figure S2A**) was tested for AMCase expression using the *E. coli* strain BL21 (DE3) as a host cell. By using the *Staphylococcus* Protein A promoter and its signal sequence, the fusion protein was designed to be constitutively expressed and secreted into periplasmic space and then the culture medium of *E. coli* [28]. Many fusion proteins purified from culture medium have already been reported by us and others [28], [33]–[35].

We first isolated the expressed fusion protein from the culture medium using IgG Sepharose and measured the chitinolytic activity. We could detect the chitinase activity in the culture medium fraction, indicating that we could successfully express the fusion protein in *E. coli*. However, the chitinase activity and the yield were quite low (Table 1). It has been shown that some His-tagged proteins can form inclusion bodies in the cytoplasm of *E. coli* [29]. Next, we examined the distribution of the chitinase activity in the periplasmic space, the soluble and insoluble fractions in *E.coli*.

**Table 1:** Subcellular distribution of chitinolytic activity in *E. coli.*

Fraction	Total activity (U)	Distribution (%)	Total Protein (mg)	Specific activity (U/mg)
Medium	0.021±0.005	4±1.0	0.208±0.07	0.107±0.038
Periplasm 1 (Peri 1)	0.201±0.063	33±6.8	0.497±0.09	0.410±0.131
Periplasm 2 (Peri 2)	0.343±0.149	54±8.5	1.03±0.11	0.349±0.164
Cytoplasm	0.044±0.009	7±1.8	0.311±0.10	0.151±0.050
Insoluble	0.014±0.009	2±1.1	0.436±0.11	0.030±0.016

The recombinant protein was prepared from the medium, periplasmic space and soluble and insoluble fractions of 1 L culture of *E. coli* as described in Materials and Methods. The preparations were performed in triplicate.

The periplasmic proteins were obtained by the osmotic shock of pelleted cells and by further treatment with lysozyme and with a second osmotic shock as described in Materials and Methods. The resulting spheroplasts were sonicated to extract soluble cytoplasmic proteins. The expressed fusion protein was isolated from these fractions using IgG Sepharose. We measured the chitinolytic activity in these soluble fractions. As shown in Table 1, more than 80% of the chitinolytic activity was detected in the periplasmic fractions [periplasmic space 1/osmotic shock (Peri 1) and periplasmic space 2/lysozyme (Peri 2) fractions] of *E. coli*. The culture medium and the cytoplasmic soluble fraction contained 5% and 10% of the total chitinolytic activity, respectively. Omission of the second osmotic shock in preparation of periplasmic space 2/lysozyme fraction (Peri 2) resulted in significant decreased chitinolytic activity in the fraction (**Table S2**), indicating that the second osmotic was effective in extracting the recombinant protein from the periplasmic space.

**Figure 2:**
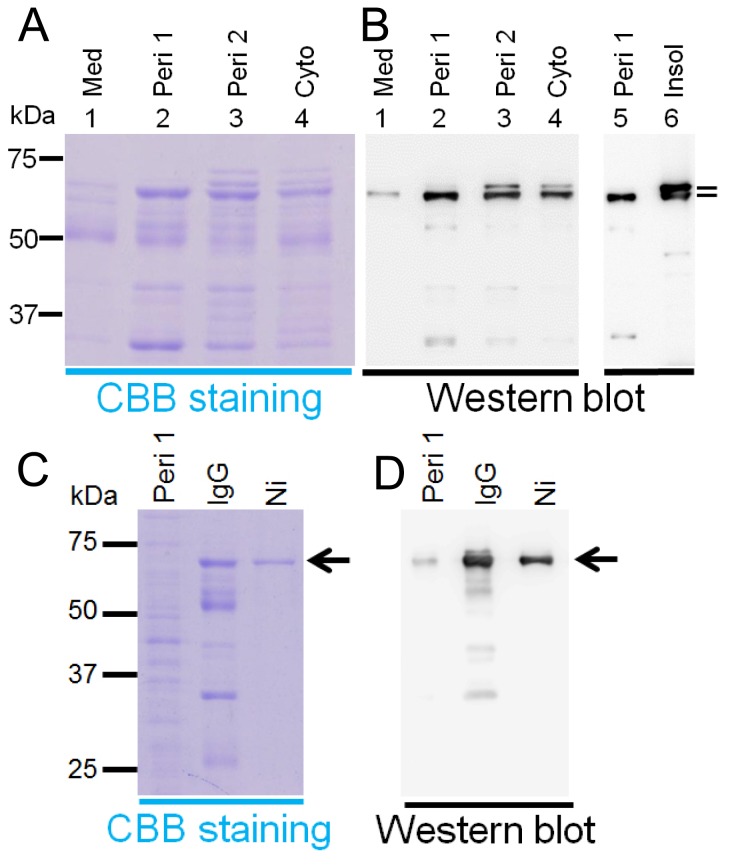
Analysis of localization of *E. coli*-produced fusion proteins. (A) 10% SDS-PAGE analysis of the recombinant proteins from the culture medium (Med), periplasmic fractions (Peri 1 and Peri 2) and cytoplasmic soluble fraction (Cyto) and the insoluble fraction (Insol) from *E. coli*. The proteins in the gel were visualized by staining with Coomassie Blue R-250. (B) Western blot analysis of the recombinant proteins. Proteins were run on SDS-PAGE and transferred to PVDF membrane. Western blots were probed with an anti-V5-HRP antibody. Approximately 2 µg of each protein was electrophoresed. The molecular mass (kDa) of the molecular weight markers (All Blue, Bio-Rad) are shown in the left margin, and the positions of the fusion proteins (Protein A-AMCase-V5-His) are shown with arrows in the right margin. (C and D) Purification of the recombinant proteins. The fusion proteins were expressed in *E. coli* and purified from the periplasmic fraction 1 (Peri 1) by IgG Sepharose followed by Ni Sepharose. Proteins separated by SDS-PAGE were stained with Coomassie Blue R-250 (C) or transferred to PVDF membrane (D). Western blots were probed with an Anti-V5-HRP antibody.

To further examine the expression of Protein A-AMCase-V5-His in *E. coli*, we analyzed the protein by SDS-PAGE. Preparations from the four compartments were electrophoresed on a 10% SDS-PAGE, which was stained with Coomassie Blue. As shown in Figure 2A, the transformed *E. coli* cells synthesized and secreted a protein of the expected size for the mature Protein A-AMCase fusion, i.e., 68 kDa in the culture medium, periplasmic and cytoplasmic fractions (**Figure S2B**). We carried out Western blotting using an anti-V5 antibody and detected a major band in both of the protein fractions from the culture medium and the periplasmic space/osmotic shock (periplasm 1), which corresponded to that for the mature Protein A-AMCase-V5-His (Figure 2B, lanes 1 and 2 and **Figure S2**B). We detected a distinct band in the periplasmic space/lysozyme (periplasm 2) and cytoplasmic fractions (Figure 2B, lanes 3 and 4). We also detected an additional band (71 kDa) that migrated slightly slower than those from the periplasm 2 and the cytoplasmic soluble fractions (Figure 2B, lanes 3 and 4). These results clearly indicate that most of the expressed chitinase activity is present as an active form in the periplasmic space fractions in *E. coli* (Figure 2 and Table 1).

Next, we examined the presence of the recombinant protein in the insoluble fraction. The “insoluble fraction” was solubilized and we performed the refolding and purification of the recombinant protein using Ni Sepharose column as described in Materials and Methods. We measured the chitinase activity of the refolded and solubilized fraction. Although we could detect the chitinolytic activity in the refolded recombinant protein from “insoluble fraction”, total chitinase activity in the insoluble fraction was very low or negligible (approximately 2%, Table 1). We also detected an additional band that migrated slightly slower than the expected band in the refolded recombinant protein fraction (Figure 2B, lanes 6). The relative abundance of the upper band was detected in the insoluble fraction when compared with the periplasmic space/osmotic shock fraction (periplasm 1) (Figure 2B, lanes 5 and 6). This may be explained by the presence of a signal peptide at the N-terminus of Protein A (**Figure S2A**), suggesting that an expressed protein with incomplete cleavage of the signal peptide (pre-Protein A-AMCase-V5-His) was present in the insoluble fraction.

### The Purification of Protein A-AMCase-V5-His by IgG Sepharose, Followed by Ni Sepharose Chromatographies

We have added affinity tails of Protein A and a His-tag to both ends of the mature AMCase (Figure 1B). Thus, the fusion protein can be further purified by both IgG Sepharose and Ni Sepharose chromatographies.

The protein present in the periplasmic fraction 1 (Peri 1) was purified by IgG Sepharose chromatography, followed by Ni Sepharose chromatography. The purified fraction was subjected to SDS-PAGE and then visualized with Coomassie Blue R-250 staining or Western blotting. The results are shown in Figure 2C and 2D. The *E. coli*-expressed fusion protein was purified from the periplasmic fraction 1 (Peri 1) by affinity chromatography at a yield of 0.5 mg fusion protein per L of culture.

### Characterization of the Protein A-AMCase-V5-His

**Figure 3:**
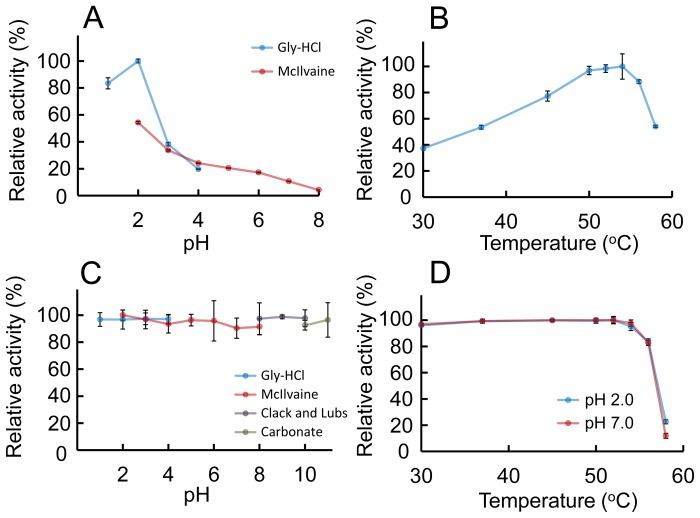
Characterization of the *E. coli*-expressed AMCase activities. (A) pH profile, (B) temperature profile, (C) pH stability profile and (D) thermostability profile of the chitinase for recombinant AMCase were measured as described in the Materials and Methods section. The values were represented as percentage of the maximum activity obtained in each series of experiments. Error bars represent the mean ± standard deviation from a single experiment conducted in triplicate.

To obtain insight into the characteristics of *E. coli*-expressed AMCase, we first examined the chitinolytic activity of the Protein A-AMCase-V5-His using 4-nitrophenyl *N,N*′-diacetyl-β-D-chitobioside as a substrate at 37°C for 30 min from pH 1.0 to 8.0. The pH optima were determined by monitoring enzyme activity at the indicated pH in 0.1 M Gly-HCl (pH 1.0 to 4.0) and McIlvaine (pH 2.0 to 8.0) buffers. As shown in Figure 3A, a striking characteristic behavioral feature of this enzyme is observed at an acidic pH. Recombinant AMCase had the highest activity at around pH 2.0 and lower activities at more neutral pH (pH 3.0∼7.0) (Figure 3A). The recombinant AMCase had properties very similar to the native enzyme from the mouse intestine regarding pH preference [14]. A stronger peak of chitinolytic activity at pH 2.0 was observed when using Gly-HCl buffer instead of McIlvaine buffer (Figure 3A).

The effect of temperature on enzyme activity was determined in 0.1 M Gly-HCl buffer at pH 2.0 at temperatures ranging from 30 to 58°C using 4-nitrophenyl *N,N*′-diacetyl-β-D-chitobioside for 15 min. As shown in Figure 3B, the rate of the recombinant AMCase-catalyzed reaction increased as the temperature increased to reach a maximum level at 54°C, then abruptly declined, indicating denaturation of the protein.

We next determined the pH stability of the recombinant AMCase. The recombinant AMCase was pre-incubated on ice for 60 min at various pH values using four different buffers (see the Materials and Methods section). After the pre-incubation, the enzyme activity was analyzed at 37°C and pH 2.0. As shown in Figure 3C, the recombinant AMCase showed remarkable acid and base stabilities. The recombinant AMCase was stable over a broad pH range (between 1.0 and 11.0), during the 1 h pre-incubation on ice. This treatment caused no measurable decrease in chitinase activity. Thus, the *E. coli-*expressed AMCase exhibited robust stability under basic as well as acidic conditions.

The thermal stability of AMCase was assessed by measuring the chitinolytic activity at elevated temperatures at pH 2.0 (optimal pH) or pH 7.0 (physiological pH). Samples were pre-incubated at the indicated pH for 20 min from 30°C to 58°C. After pre-incubation, we measured the residual activity against 4-nitrophenyl *N,N*′-diacetyl-β-D-chitobioside at pH 2.0. As shown in Figure 3D, recombinant AMCase was heat-stable till 54°C, both at pH 2.0 and 7.0, respectively. Under these conditions, the enzyme showed a decrease in chitinolytic activity at temperatures above 56°C. These results indicated that recombinant AMCase is heat stable both in acidic and neutral conditions.

### Chitinolytic Activity of Recombinant AMCase Comparable to CHO-expressed One

We next evaluated chitin hydrolytic activities of *E. coli*-expressed Protein A-AMCase-V5-His by comparing that with CHO-expressed AMCase-V5-His. Since we prepared the CHO-expressed protein by Ni resin, it contained several proteins other than the target. In addition, it is possible that some part of the *E. coli*-expressed protein contained misfolding protein. The impurity or misfolding of enzymes may cause confusion when comparing specific activity between the two enzyme preparations. For the elimination of the errors, we first determined the chitinolytic activity of the mouse AMCase preparations. Additionally, we performed Western blots of the mouse AMCase preparation.

**Figure 4:**
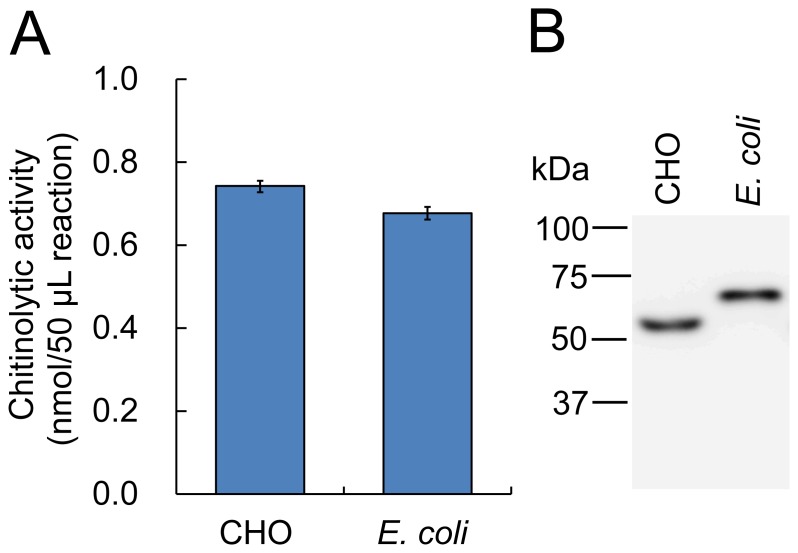
Comparison of the chitinolytic properties of murine AMCase prepared from *E. coli* with the enzyme from CHO cells. We first measured the chitinolytic activity of the enzyme preparations from CHO cells and *E. coli* and by using page in a volume of 50 µL in 0.1 M Gly-HCl buffer (pH 2.0) at 37°C for 30 min. Then we adjusted the enzyme solutions to give rise to the same activity (A). We analyzed the immunoreactivities of these enzymes by Western blot using an anti-V5 antibody, which recognized both recombinant AMCase proteins (B). The enzyme fractions with the same chitinase activities were visualized via SDS-PAGE, followed by Western blotting using an anti-V5 antibody.

We first measured the chitinolytic activity of the enzyme preparations by using 4-nitrophenyl *N,N*′-diacetyl-β-D-chitobioside and adjusted the enzyme solutions to give rise to the same activity (Figure 4A). Then, we analyzed the immunoreactivities of these enzymes by Western blot using an anti-V5 antibody, which recognized the recombinant AMCase fusion proteins produced in CHO and in *E. coli* (Figure 1). The enzyme fractions with the same chitinase activities were run on an SDS-PAGE gel, followed by Western blotting using an anti-V5 antibody. We expressed mouse AMCase as the mature AMCase-V5-His in CHO cells and the mature Protein A-AMCase-V5-His in *E. coli* (**Figure S1B** and **Figure S2B**). As shown in Figure 4B, molecular mass of Protein A-AMCase-V5-His expressed in *E. coli* was higher than that of AMCase-V5-His. CHO-expressed AMCase and *E. coli*-produced AMCase gave similar signals in the immunoblot analysis, which are approximately 54 kDa and 68 kDa, respectively. The difference in the molecular mass of the proteins obtained from CHO cells and *E coli* was due to the presence of Protein A region in the protein obtained from *E. coli* (**Figure S1B** and **Figure S2B**). We could show that there is an experimental equivalence between the CHO-expressed AMCase-V5-His and *E. coli* expressed Protein A-AMCase-V5-His.

### Protein A-AMCase-V5-His Facilitates Chitin Binding and Promotes Chitin Degradation

**Figure 5:**
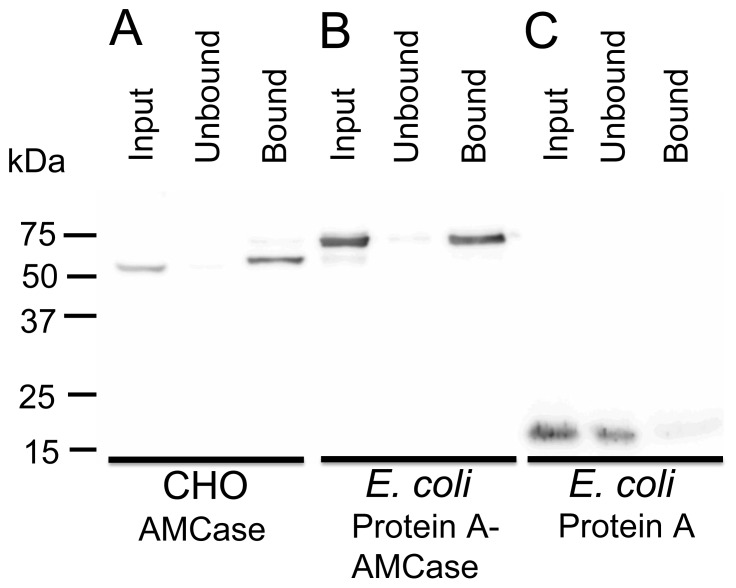
Binding analysis of CHO-expressed or *E. coli*-produced AMCase to chitin beads. (A) CHO-expressed AMCase-V5-His, (B) *E. coli*-produced Protein A-AMCase-V5-His, (C) *E. coli*-produced Protein A. Chitin-binding assays using chitin beads were carried out as described in the Materials and Methods section. The recombinant fusion with the chitin-binding domain (CBD) of AMCase bound to the chitin beads (A and B), and the fusion protein without the chitin-binding domain bound to the chitin beads (C), indicating that the recombinant chitin-binding domain bound to chitin.

The expressed fusion protein contains a chitin-binding domain at the C-terminus of AMCase region (Figure 1). To determine whether the chitin-binding domain (CBD) in the recombinant protein is functionally active, we carried out a binding assay using chitin beads (see the Materials and Methods section). In this assay, chitinase that is capable of binding to chitin beads was precipitated by incubation and subsequent centrifugation. As shown in Figure 5, most of the fusion protein of CHO-expressed mature AMCase-V5-His or *E. coli*-produced mature Protein A-AMCase-V5-His was detected in the chitin beads bound fraction. In contrast, fusion proteins without the AMCase region (mature Protein A-V5-His, **Figure S2D**) were present in the supernatant (unbound fraction). These data indicated that the recombinant AMCase can bind to chitin.

**Figure 6:**
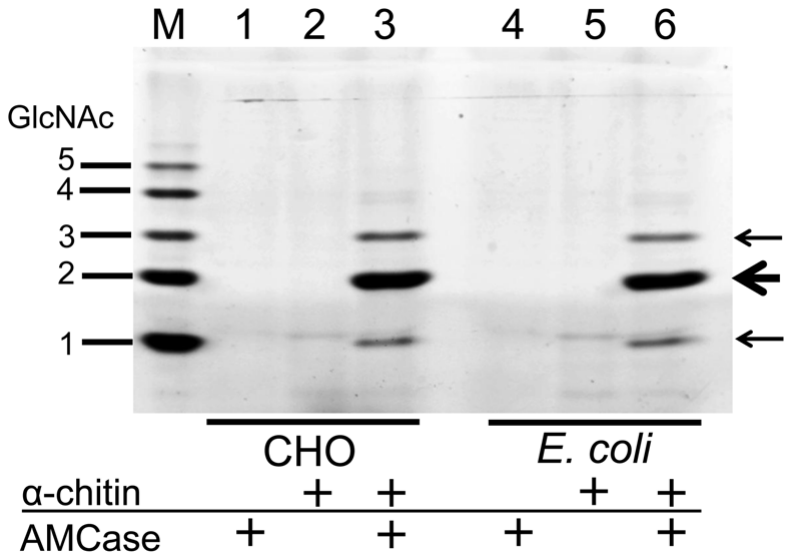
Degradation products of colloidal chitin by CHO- and *E. coli*-expressed mouse AMCase. Colloidal chitin was used as a substrate to determine the chitinase activity of CHO-expressed or *E. coli*-expressed protein in 0.1 M Gly-HCl buffer. Reactions were conducted for 1 h at 37°C. The chitin fragments generated by the recombinant AMCase proteins were analyzed by fluorophore-assisted carbohydrate electrophoresis [14], [32]. Chitin oligomers are shown in the left margin. Fluorophore-assisted carbohydrate electrophoresis analysis revealed that the recombinant mouse AMCase releases mainly (GlcNAc)_2_ fragments from chitin.

It has been reported that human recombinant Chit1 and mouse recombinant AMCase are able to degrade colloidal chitin and give rise to a dimer oligosaccharide [14]. Finally, we incubated the colloidal chitin with the CHO- and *E. coli*-expressed AMCase proteins. The resulting monosaccharide and oligosaccharides were labeled covalently at their reducing end groups with the fluorophore and the resulting fluorescent derivatives were separated by high-resolution PAGE, as described previously [32]. As shown in Figure 6, both CHO- and *E. coli-*expressed mouse AMCase proteins released mainly (GlcNAc)_2_ fragments and the GlcNAc monomer from colloidal chitin, which are consistent with the products of human recombinant Chit1 and mouse recombinant AMCase expressed in COS-1 cells [14]. Taken together, these results indicate that *E. coli*-expressed AMCase can be considered to be a functional enzyme comparable to CHO-expressed AMCase.

## Discussion

AMCase may play important roles in asthma, immune response and food processing. Little is known, however, about the pathophysiological functions of AMCase in mice and humans. Large quantities of the functional protein are required for biochemical characterization of AMCase. This necessitates the use of an expression system that is simple, rapid and inexpensive. *E. coli* overexpression systems are widely used for this purpose because *E. coli* grows quickly in an inexpensive medium and can be easily scaled up for production. Here, we described an *E. coli*-expression system that allows for the periplasmic production of mouse AMCase with chitinolytic activity comparable to a cultured cell-expressed AMCase.

The mouse AMCase was expressed as a fusion protein with Protein A, a V5 epitope and a (His)_6_ tag (V5-His)(Figure 1) using the pEZZ18 vector [28]. This is a Protein A gene fusion vector system based on two synthetic IgG-binding domains (ZZ) of *Staphylococcus aureus* Protein A, which has been used for extracellular expression of secretory proteins and for short proteins [28], [33]–[35]. Expression of the fusion protein is controlled by the *Staphylococcus aureus* Protein A promoter, which is not inducible. Because the pEZZ18 contains a signal sequence of *Staphylococcus* Protein A, expressed fusion proteins are secreted into aqueous culture medium under the direction of the signal sequence. The *E. coli* expression system was capable of producing a functional AMCase. In our case, most of the expressed Protein A-AMCase-V5-His was present in periplasmic fraction of *E. coli* (Figure 2 and Table 1).

The recombinant protein showed profound acid stability at pH 1 to 3 (Figure 3C). Thus, we could use IgG Sepharose as an affinity chromatography resin for purifying the Protein A-fusion protein. The soluble gene fusion product can be rapidly recovered in a one-step procedure by IgG affinity chromatography. The bound protein should be eluted with 0.1 M Gly-HCl (pH 2.5). This method can only be used if the fusion product is stable under these conditions. Our results clearly show that the pEZZ18 system is the best fit for the expression of mouse AMCase, which is an acid-stable secretory enzyme.

The objective of the studies described here was to compare the enzymatic properties of murine AMCase prepared from *E. coli* with the enzyme from CHO cells. N-terminal or C-terminal of His tags are included for purification purposes in expression of AMCase using the insect or mammalian cultured cell system [18], [19], [23]–[25], [27]. Regarding the optimal pH and acid stability, the enzymatic characteristics of the *E. coli*-expressed AMCase are consistent with the native chitinase data. In addition, recombinant AMCase facilitates chitin binding. Furthermore, recombinant AMCase degraded colloidal chitin and produced mainly *N,N′*-diacetylchitobiose. Thus, *E. coli*-expressed AMCase showed properties similar to the native enzyme from mice [14] or CHO-expressed AMCase.

Because of the unique folding properties of the 17.8 kDa Protein A, this protein had little effect on the folding of the fusion partner into a native conformation. Because *E. coli*-expressed AMCase had properties similar to the native enzyme found in the mouse intestine and CHO-expressed AMCase, AMCase expressed in the periplasmic space of *E. coli* tended to form an active tertiary structure identical to that of the naturally synthesized mouse AMCase. Our results clearly indicate that the primary structure of AMCase is strong enough to form a proper tertiary structure for chitinolytic activity. The formation of this tertiary structure may be due to the conserved sequence among ancient chitinase family [6] and/or periplasmic expression.

This expression system for mouse AMCase has several important advantages. First, most of the Protein A-fusion protein was present as a periplasmic soluble protein, although a small part was present in the intracellular and insoluble fractions in which enzyme activity after the refolding procedure was negligible. Second, pEZZ18 vector uses the *Staphylococcus aureus* Protein A promoter, which is not inducible and thus inexpensive. Although pEZZ18 Protein A promoter activity is not as robust as T7, we obtained an active enzyme by overnight culture without IPTG (isopropyl-β-thiogalactopyranoside) induction. We could obtain sufficient amounts of the recombinant AMCase for further biochemical analysis. When we need more protein, we can easily increase *E. coli* culture volume. Finally, we can remove the Protein A-AMCase-V5-His from the reaction mixture easily by passing the protein through an IgG Sepharose column or a Ni Sepharose column after incubation with several types of chitins.

We recently reported that AMCase mRNA is synthesized at extraordinarily high levels in the mouse stomach [20], [21]. Recombinant mouse AMCase is most active at pH 2.0, which reflects the stomach’s acidity and shows profound acid stability (Figure 3A and 3C). This result is consistent with previous observations using native enzymes from the mouse intestine and stomach [14], [21]. The unusual acid dependence and stability of the mouse AMCase in acidic conditions allow the efficient digestion of chitinous materials under the extreme acidic environment in the stomach.

The mouse AMCase is more active in Gly-HCl buffer than in McIlvaine at pH 2.0 (Figure 3A). The reason for this result is not well understood, but the following possibilities should be considered. Pepsin is expressed as a pro-form zymogen, pepsinogen. Hydrochloric acid is secreted in the stomach, creating acidic conditions (pH = ∼2), which allow pepsinogen to unfold and cleave itself in an autocatalytic fashion, thereby generating the active form of pepsin [36], [37]. Although AMCase is not synthesized as a pro-form present, hydrochloric acid may induce similar activation for AMCase in stomach.

Chitinases cleave chitin polymers into oligosaccharides of varying sizes (endochitinase activity) and release glucosamine monosaccharide from the end of a chitin polymer (exochitinase activity) [38]–[40]. Recently Eide et al. reported that human Chit1 degrades chitosan, which is a linear polysaccharide composed of randomly distributed β-(1–4)-linked D-glucosamine and N-acetyl-D-glucosamine, primarily via an endoprocessive mechanism [41]. The properties how AMCase acts on chitin and chitosan have not been studied previously. AMCase has been implicated in the pathophysiology of allergic airway diseases [16]–[19]. We will carry out detailed analysis of the enzymatic properties of our recombinant AMCase on chitin and chitosan.

Mouse AMCase may play important physiological roles in nutrition and defense. Furthermore, the haplotype encoding a specific variant is associated with protection from asthma in several ethnic populations [17]. A genetic variant in the human AMCase gene results in AMCase variants that modulate the enzymatic activity of AMCase [18]. Using the site-directed mutagenesis of AMCase expressed in COS-7 cells, Bussink et al. have shown that His187 is responsible for the acidic optimum pH [22]. The *E. coli*-produced recombinant protein may be a valuable tool for elucidating biological functions of the enzyme and performing a detailed structure and functional relationship analysis. Because inhibition of AMCase has been suggested as a therapeutic strategy against asthma [16], [25], the substrate specificity and analysis of the product using the recombinant AMCase reported in this study are of medical interest.

## Supporting Information

Figure S1 **Deduced amino acid sequences and their theoretical molecular masses of pre-AMCase-V5-His and mature-AMCase-V5-His.** The amino acid sequences are color coded, consistent with Figure 1A. Blue, signal sequence of mouse AMCase; Pink, mouse mature AMCase; Green, V5-His sequence.
(DOC)

Figure S2 **Deduced amino acid sequences and their molecular masses of recombinant proteins expressed in** ***E. coli*** **.** The amino acid sequences are color coded, consistent with Figure 1B and 1C. Rich blue, signal sequence of Protein A; Yellow, truncated form of Protein A; Pink, mouse mature AMCase; Green, V5-His sequence.
(DOC)

Table S1 **Forward and reverse primers used to construct the mammalian and** ***E. coli*** **-expression vectors.**
(DOC)

Table S2 **Effect of omission of the second osmotic shock on the distribution of chitinolytic activity.** The recombinant protein was prepared from 0.5 L culture of *E. coli.* The periplasmic space 2/lysozyme fraction (Peri 2) was prepared as described in Materials and Methods without the second osmotic shock. The preparations were performed in triplicate.
(DOC)
