# Supplementary material for: Protein A-Mouse Acidic Mammalian Chitinase-V5-His Expressed in Periplasmic Space of Escherichia coli Possesses Chitinase Functions Comparable to CHO-Expressed Protein
Source: PLoS One. 2013 Nov 11;8(11):e78669. doi: 10.1371/journal.pone.0078669 (PMC3823863; doi:10.1371/journal.pone.0078669)
Supplement: Figure S1 — Deduced amino acid sequences and their theoretical molecular masses of pre-AMCase-V5-His and mature-AMCase-V5-His. The amino acid sequences are color coded, consistent with Figure 1A. Blue, signal sequence of mouse AMCase; Pink, mouse mature AMCase; Green, V5-His sequence. (DOC) [file pone.0078669.s001.doc]

1. **Pre-AMCase-V5-His**

**503 amino acids 55,339 dalton**

**MAKLLLVTGLALLLNAQLGSAYNLICYFTNWAQYRPGLGSFKPDDINPCLCTHLIYAFAGMQNNEITTIEWNDVTLYKAFNDLKNRNSKLKTLLAIGGWNFGTAPFTTMVSTSQNRQTFITSVIKFLRQYGFDGLDLDWEYPGSRGSPPQDKHLFTVLVKEMREAFEQEAIESNRPRLMVTAAVAGGISNIQAGYEIPELSKYLDFIHVMTYDLHGSWEGYTGENSPLYKYPTETGSNAYLNVDYVMNYWKNNGAPAEKLIVGFPEYGHTFILRNPSDNGIGAPTSGDGPAGPYTRQAGFWAYYEICTFLRSGATEVWDASQEVPYAYKANEWLGYDNIKSFSVKAQWLKQNNFGGAMIWAIDLDDFTGSFCDQGKFPLTSTLNKALGISTEGCTAPDVPSEPVTTPPGSGSGGGSSGGSSGGSGFCADKADGLYPVADDRNAFWQCINGITYQQHCQAGLVFDTSCNCCNWPARGHPFEGKPIPNPLLGLDSTRTGHHHHHH**

1. **Mature AMCase-V5-His**

**482 amino acids 53,246 dalton**

**YNLICYFTNWAQYRPGLGSFKPDDINPCLCTHLIYAFAGMQNNEITTIEWNDVTLYKAFNDLKNRNSKLKTLLAIGGWNFGTAPFTTMVSTSQNRQTFITSVIKFLRQYGFDGLDLDWEYPGSRGSPPQDKHLFTVLVKEMREAFEQEAIESNRPRLMVTAAVAGGISNIQAGYEIPELSKYLDFIHVMTYDLHGSWEGYTGENSPLYKYPTETGSNAYLNVDYVMNYWKNNGAPAEKLIVGFPEYGHTFILRNPSDNGIGAPTSGDGPAGPYTRQAGFWAYYEICTFLRSGATEVWDASQEVPYAYKANEWLGYDNIKSFSVKAQWLKQNNFGGAMIWAIDLDDFTGSFCDQGKFPLTSTLNKALGISTEGCTAPDVPSEPVTTPPGSGSGGGSSGGSSGGSGFCADKADGLYPVADDRNAFWQCINGITYQQHCQAGLVFDTSCNCCNWPARGHPFEGKPIPNPLLGLDSTRTGHHHHHH**
