# Supplementary material for: Protein A-Mouse Acidic Mammalian Chitinase-V5-His Expressed in Periplasmic Space of Escherichia coli Possesses Chitinase Functions Comparable to CHO-Expressed Protein
Source: PLoS One. 2013 Nov 11;8(11):e78669. doi: 10.1371/journal.pone.0078669 (PMC3823863; doi:10.1371/journal.pone.0078669)
Supplement: Figure S2 — Deduced amino acid sequences and their molecular masses of recombinant proteins expressed in E. coli . The amino acid sequences are color coded, consistent with Figure 1B and 1C. Rich blue, signal sequence of Protein A; Yellow, truncated form of Protein A; Pink, mouse mature AMCase; Green, V5-His sequence. (DOC) [file pone.0078669.s002.doc]

1. **Pre-Protein A-AMCase-V5-His**

**645 amino acids 71,255 dalton**

**MKKKNIYSIRKLGVGIASVTLGTLLISGGVTPAANAAQHDEAVDNKFNKEQQNAFYEILHLPNLNEEQRNAFIQSLKDDPSQSANLLAEAKKLNDAQAPKVDNKFNKEQQNAFYEILHLPNLNEEQRNAFIQSLKDDPSQSANLLAEAKKLNDAQAPKVDANSYNLICYFTNWAQYRPGLGSFKPDDINPCLCTHLIYAFAGMQNNEITTIEWNDVTLYKAFNDLKNRNSKLKTLLAIGGWNFGTAPFTTMVSTSQNRQTFITSVIKFLRQYGFDGLDLDWEYPGSRGSPPQDKHLFTVLVKEMREAFEQEAIESNRPRLMVTAAVAGGISNIQAGYEIPELSKYLDFIHVMTYDLHGSWEGYTGENSPLYKYPTETGSNAYLNVDYVMNYWKNNGAPAEKLIVGFPEYGHTFILRNPSDNGIGAPTSGDGPAGPYTRQAGFWAYYEICTFLRSGATEVWDASQEVPYAYKANEWLGYDNIKSFSVKAQWLKQNNFGGAMIWAIDLDDFTGSFCDQGKFPLTSTLNKALGISTEGCTAPDVPSEPVTTPPGSGSGGGSSGGSSGGSGFCADKADGLYPVADDRNAFWQCINGITYQQHCQAGLVFDTSCNCCNWPARGHPFEGKPIPNPLLGLDSTRTGHHHHHH**

1. **Mature Protein A-AMCase-V5-His**

**609 amino acids 67,629 dalton**

**AQHDEAVDNKFNKEQQNAFYEILHLPNLNEEQRNAFIQSLKDDPSQSANLLAEAKKLNDAQAPKVDNKFNKEQQNAFYEILHLPNLNEEQRNAFIQSLKDDPSQSANLLAEAKKLNDAQAPKVDANSYNLICYFTNWAQYRPGLGSFKPDDINPCLCTHLIYAFAGMQNNEITTIEWNDVTLYKAFNDLKNRNSKLKTLLAIGGWNFGTAPFTTMVSTSQNRQTFITSVIKFLRQYGFDGLDLDWEYPGSRGSPPQDKHLFTVLVKEMREAFEQEAIESNRPRLMVTAAVAGGISNIQAGYEIPELSKYLDFIHVMTYDLHGSWEGYTGENSPLYKYPTETGSNAYLNVDYVMNYWKNNGAPAEKLIVGFPEYGHTFILRNPSDNGIGAPTSGDGPAGPYTRQAGFWAYYEICTFLRSGATEVWDASQEVPYAYKANEWLGYDNIKSFSVKAQWLKQNNFGGAMIWAIDLDDFTGSFCDQGKFPLTSTLNKALGISTEGCTAPDVPSEPVTTPPGSGSGGGSSGGSSGGSGFCADKADGLYPVADDRNAFWQCINGITYQQHCQAGLVFDTSCNCCNWPARGHPFEGKPIPNPLLGLDSTRTGHHHHHH**

1. **Pre-Protein A-V5-His**

**193 amino acids 21,362 dalton**

**MKKKNIYSIRKLGVGIASVTLGTLLISGGVTPAANAAQHDEAVDNKFNKEQQNAFYEILHLPNLNEEQRNAFIQSLKDDPSQSANLLAEAKKLNDAQAPKVDNKFNKEQQNAFYEILHLPNLNEEQRNAFIQSLKDDPSQSANLLAEAKKLNDAQAPKVDANSARGHPFEGKPIPNPLLGLDSTRTGHHHHHH**

1. **Mature Protein A-V5-His**

**157 amino acids 17,737 dalton**

**AQHDEAVDNKFNKEQQNAFYEILHLPNLNEEQRNAFIQSLKDDPSQSANLLAEAKKLNDAQAPKVDNKFNKEQQNAFYEILHLPNLNEEQRNAFIQSLKDDPSQSANLLAEAKKLNDAQAPKVDANSARGHPFEGKPIPNPLLGLDSTRTGHHHHHH**
