# Supplementary material for: Protein A-Mouse Acidic Mammalian Chitinase-V5-His Expressed in Periplasmic Space of Escherichia coli Possesses Chitinase Functions Comparable to CHO-Expressed Protein
Source: PLoS One. 2013 Nov 11;8(11):e78669. doi: 10.1371/journal.pone.0078669 (PMC3823863; doi:10.1371/journal.pone.0078669)
Supplement: Table S1 — Forward and reverse primers used to construct the mammalian and E. coli -expression vectors. (DOC) [file pone.0078669.s003.doc]

EcoRI-pre-AMCase-Fw

5’-**CATG**GAATTCCGGGAGGAACGATGGCCAAGCTACT-3’

XhoI-pre-AMCase-Rv

5’-**GTGAC**CTCGAGCTGGCCAGTTGCAGCAATTACAGC-3’

EcoRI-mature-AMCase-Fw

5’-**CATG**GAATTCGTACAATCTGATATGCTATTTCACC-3’

SalI-pcDNA BGH-Rv

5’-**AGGGG**TCGACTAGAAGGCACAGTCGAGGCTGATCA-3’
