# Supplementary material for: Protein A-Mouse Acidic Mammalian Chitinase-V5-His Expressed in Periplasmic Space of Escherichia coli Possesses Chitinase Functions Comparable to CHO-Expressed Protein
Source: PLoS One. 2013 Nov 11;8(11):e78669. doi: 10.1371/journal.pone.0078669 (PMC3823863; doi:10.1371/journal.pone.0078669)
Supplement: Table S2 — Effect of omission of the second osmotic shock on the distribution of chitinolytic activity. The recombinant protein was prepared from 0.5 L culture of E. coli. The periplasmic space 2/lysozyme fraction (Peri 2) was prepared as described in Materials and Methods without the second osmotic shock. The preparations were performed in triplicate. (DOC) [file pone.0078669.s004.doc]

|  |  |  |
| --- | --- | --- |
| **Fraction** | **Total activity (U)** | **Distribution (%)** |
| Medium | 0.017 ± 0.001 | 5 ± 0.7 |
| Periplasm 1 (Peri 1) | 0.129 ± 0.011 | 38 ± 5.0 |
| Periplasm 2 (Peri 2) | 0.045 ± 0.002 | 13 ± 1.8 |
| Cytoplasm | 0.143 ± 0.035 | 41 ± 6.7 |
| Insoluble | 0.008 ± 0.001 | 2 ± 0.1 |
